# A chemical signal that promotes insect survival via thermogenesis

**DOI:** 10.21203/rs.3.rs-2756320/v1

**Published:** 2023-05-11

**Authors:** Lilin Zhao, Jiao Zhou, Junxian Chen, Xueying Zhang, Hongxia Zhang, lu guo, Defeng Li, Jing Ning, Xinchen Wang, Wanzhu Jin, Kevin Mai, Elijah Abraham, Rebecca Butcher, Jianghua Sun

**Affiliations:** Institute of Zoology, Chinese Academy of Sciences; Institute of Zoology, Chinese Academy of Sciences; Institute of Zoology, Chinese Academy of Sciences; Institute of Zoology, Chinese Academy of Sciences; Institute of Zoology, Chinese Academy of Sciences; Institute of Microbiology, Chinese Academy of Sciences; Institute of Zoology, Chinese Academy of Sciences; Institute of Zoology, Chinese Academy of Sciences; Institute of Zoology, Chinese Academy of Sciences; Department of Chemistry, University of Florida; Department of Chemistry, University of Florida; University of Florida; Institute of Zoology

## Abstract

Cold-activated thermogenesis of brown adipose tissues (BAT) is vital for the survival of animals under cold stress and also inhibits the development of tumours. The development of small-molecule tools that target thermogenesis pathways could lead to novel therapies against cold, obesity, and even cancer. Here, we identify a chemical signal that is produced in beetles in the winter to activate fat thermogenesis. This hormone elevates the basal body temperature by increasing cellular mitochondrial density and uncoupling in order to promote beetle survival. We demonstrate that this hormone activates UCP4- mediated uncoupled respiration through adipokinetic hormone receptor (AKHR). This signal serves as a novel fat-burning activator that utilizes a conserved mechanism to promote thermogenesis not only in beetles, nematode and flies, but also in mice, protecting the mice against cold and tumor growth. This hormone represents a new strategy to manipulate fat thermogenesis.

## Introduction

Generating heat and maintaining body temperature are critical for winter survival and tumour suppression in animals, particularly in mammals^1–3^. They arrest their development and stop food consumption during hibernation, and instead use stored fat to sustain their energetic demands^4^. Cold- induced sympathetic activation induces brown adipose tissue (BAT) thermogenesis and conversion of white adipocytes to brown/beige adipocytes^5,6^. These activated brown/beige adipocytes generate heat through non-shivering thermogenesis, which is mediated by mitochondrial biogenesis and the uncoupling of ATP production in the inner membrane of the mitochondria^7–10^. Ad7ipocytes have even been found to directly sense and elevate temperature through a thermogenic transcriptional response in BAT^11^. The molecular mechanisms of cold-activated thermogenesis have been studied^12^. However, the upstream signals that mediate mitochondrial biogenesis and uncoupling for thermogenesis in fat tissues are not fully understood.

Ascarosides are best known for their role as pheromones that induce developmental arrest, increase stress resistance, and modulate behavior in a variety of nematodes species^13^. These pheromones were first identified in the free-living nematode *Caenorhabditis elegans*, where they induce the dauer larval stage, which does not feed but instead lives off of fat stores^14^. Since then, it has been shown that a variety of parasitic nematodes produce ascarosides^15^. The plant-parasitic pinewood nematode secretes ascarosides (specifically asc-C5) to promote the pupation of its vector beetle, *Monochamus alternutus*, which carries the nematode between pine trees^16^. Furthermore, we showed that ascarosides are not limited to nematodes and are produced by the *M. alternutus* beetle as well. The beetle larvae produce ascarosides (specifically asc-C9) that induce developmental arrest of the beetle and increase the cold hardiness of the larvae in the winter^16,17^. These findings suggest that ascarosides may have analogous roles in nematodes and beetles and led us to investigate the mechanism whereby ascarosides improve the survival of the beetle under cold conditions.

Here, we show that cold exposure induces the formation of *M. alternatus* beige-type larvae, which have markedly improved survival over white-type larvae. Beige-type larvae possess enhanced mitochondrial biogenesis and uncoupling ability that promotes temperature maintenance and survival. The production of ascaroside C9 is induced in beige-type larvae in the winter months and is a key signal that induces thermogenesis in subcutaneous fat body cells in *M. alternatus*. Specifically, this activation of thermogenesis occurs through AKHR and depends on UCP4, which activates mitochondrial uncoupling, and PGC1α, which stimulates an increase in mitochondrial density. Importantly, the function of this hormone is conserved through evolution in nematodes, flies, and even mice, where it can be used to induce browning of white adipose tissue (WAT) and BAT activation through UCP1.

### Cold-induced thermogenesis of beige-type larvae

**Figure 1:**
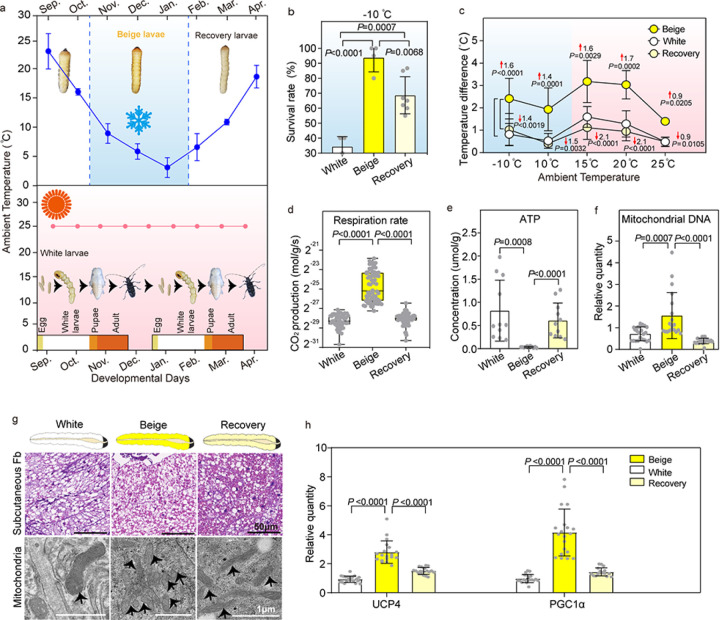
Thermogenesis of beige-type larvae under cold stress. **a.** Body color of beige-type larvae (fifth-stage larvae that had experienced low temperature), recovery-type larvae (fifth-stage larvae that had experienced warm temperature), and white-type larvae (fifth-stage larvae that had been sustained at 25°C in the laboratory for 25 generations). The outside temperature from September to April of 2018–2022 in Nanjing province was obtained from agriculture and agrometeorological data system (XMY1.4.2b). The white-type beetle (sustained at 25°C in the laboratory) experienced 2 generations during the fifth larval stage period of beige- and recovery-type larvae in the field. **b.** Survival of white-, beige- and recovery-type larvae that experienced −10 °C for 17h and then were transferred to 25°C for 24h (n=4 with 20 beetles per group). **c.** The temperature difference between the body surface and ambient temperature when the larvae were transferred through a temperature gradient from −10 °C to 10 °C, then to 15^°o^C, 20°C and 25°C (n=5 with 20 beetles per group). **d-f.** The respiration rate, ATP concentration, and the mitochondrial number in white-, beige- and recovery- type larvae (n=3 with 10 beetles per group). **g.** Lipid droplets in hematoxylin and eosin-stained sections of the subcutaneous fat body and mitochondria of white-, beige- and recovery-type larvae (n = 3 with 3 beetles per group). **h.** Relative expression of UCP4 and PGC1α in white-, beige- and recovery-type larvae (normalized to b-actin) (n=3 with 10 beetles per group).

To characterize the overwintering developmental arrest, we analyzed the physiological characteristics of fifth stage beetle larvae in the field during the fall, winter, and spring months. In the field, the larvae exposed to cold stress during the winter from early November to late January are referred to here as beige-type larvae based on their yellow body color (Fig. 1a). The body color of beige-type larvae changed to white when the temperature began to rise from February to middle April, and these larvae are referred to as recovery-type larvae (Fig. 1a). In contrast, when the beetles were maintained at 25°C in the laboratory, the larvae had a white body color and are referred to as white-type larvae (Fig. 1a). The white- type larvae quickly molt to pupae in 10–15 days in the lab while beige-type larvae remain as fifth stage larvae for nearly 90 days in the field (Fig. 1a). Beige-type larvae are resistant to cold shock as 93.75% of the beige-type larvae survived after 17h of cold shock at −10°C, whereas 68.63% and 34.32% of the recovery-type and white-type larvae, respectively, survived (Fig. 1b). After 24h of recovery from cold shock at 25°C, the beige-type larvae became active more quickly than recovery- and white-type larvae (Fig. S1a).

To assess the energy metabolism during cold shock, we monitored body temperature maintenance in the three types of larvae under cold exposure. The body temperature of beige-type larvae was significantly higher than white- and recovery-type larvae at −10°C. After the cold exposure, they were either gradually warmed to 25 °C or immediately shifted to 25°C. The beige-type larvae recovered more rapidly to ambient temperature with higher body temperature than white- or recovery- type larvae (Fig. 1c, Fig. S1b, S1c).

Importantly, beige-type larvae showed a higher respiration rate, lower ATP concentration (Fig. 1d, e), and increased mitochondrial density (Fig. 1f, g). These suggested that these larvae used enhanced respiration and mitochondrial uncoupling to increase thermogenesis and cold shock survival^6^. Furthermore, the transcription of genes involved in lipid metabolism and respiration were up-regulated in the beige-type larvae (Fig. S1d). The decreased triacyglycerol (TAG) content, smaller lipid droplets, and decreased free fatty acids (Fig. 1g, Fig. S1e, f, g) in the beige-type larvae indicated lipolysis was stimulated. Consistent with the metabolic shift seen in beige-type larvae, peroxisome proliferator-activated receptor gamma coactivator 1-α (PGC1α), a master regulator of mitochondrial biogenesis, and mitochondrial uncoupling protein 4 (UCP4) were upregulated in beige-type larvae (Fig. 1h).

To investigate the mechanism of increased respiration in beige-type larvae, beige- and white-type larvae were injected with oligomycin, which blocks ATP synthase, or stearic acid, which induces mitochondrial uncoupling^18^. With oligomycin treatment, beige-type larvae still had higher respiration rates than white- type larvae (Fig. S1h). Stearic acid did not increase the respiration of beige-type larvae, suggesting that mitochondrial uncoupling is occurring in beige-type larvae and thus cannot be further enhanced pharmacologically (Fig. S1i). Furthermore, guanosine triphosphate (GTP), which reduces uncoupled respiration^18,19^, decreased the respiration rates of beige-type larvae (Fig. S1j). These results suggest that increased respiration of beige-type larvae is independent of mitochondrial ATP synthesis and is induced through mitochondrial uncoupling.

### Asc-C9-induced thermogenesis in subcutaneous fat body cells improves survival

**Figure 2:**
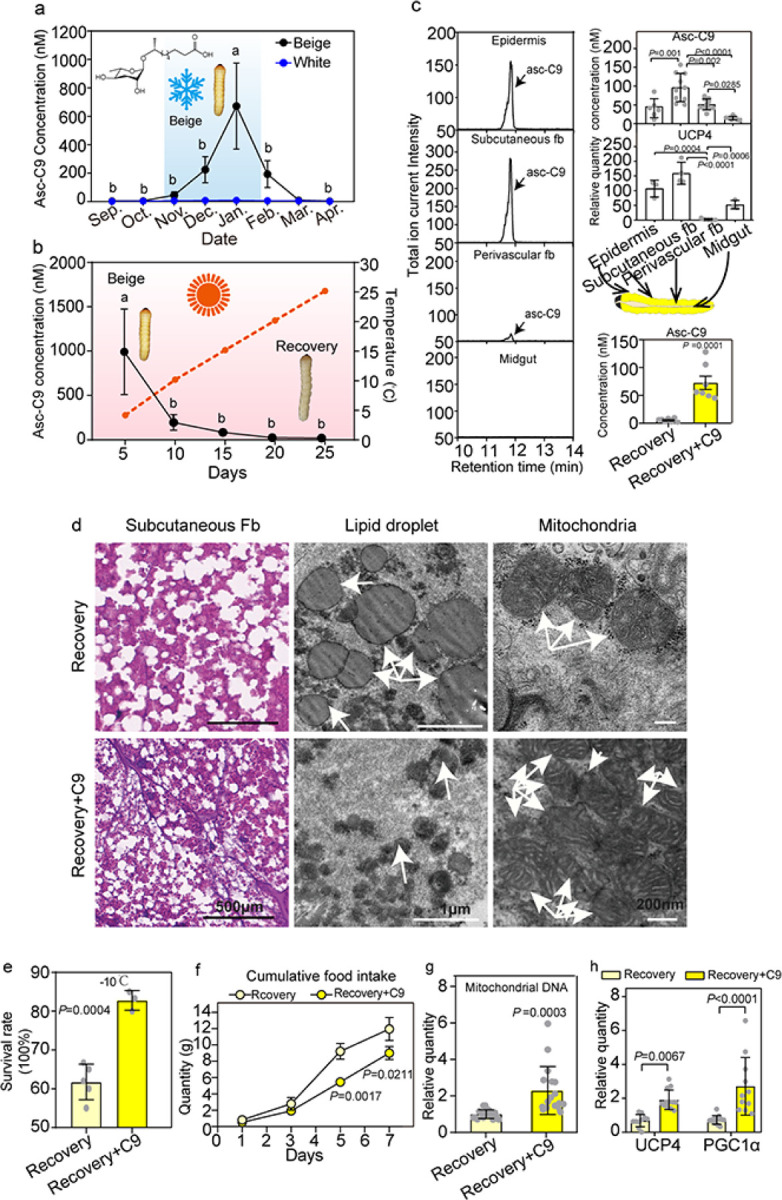
Asc-C9 induces thermogenesis of subcutaneous fat body. **a.** The changes in asc-C9 concentration in fifth-stage larvae during the overwintering period (n=4 with more than 20 beetles per larval type and time point). **b.** Effect of temperature on the production of asc-C9 in fifth-stage larvae (n=4 with more than 20 beetles per temperature point). **c.** Asc-C9 content and expression of UCP4 in different tissues of beige-type larvae (n=4 with 10 beetles per group). The asc-C9 concentration after injection in recovery-type larvae (n=3 with 10 beetles per group). **d.** Lipid droplets in hematoxylin-eosin stained sections and transmission electron microscopy of mitochondria in subcutaneous fat body of recovery-type larvae injected with either vehicle or asc-C9 (n=3 with 3 beetles per group). (e-g) Recovery-type larvae as control groups; recovery-type larvae injected with asc-C9 as treatment groups. **e.** Survival rate at −10°C of recovery-type larvae injected with either vehicle or asc-C9 (n=3 with 10 beetles per group). **f.** Cumulative food intake at 25°C for 7 days of recovery-type larvae injected with either vehicle or asc-C9 (n=3 with 20 beetles per group). **g.** The relative quantity of mitochondrial DNA in recovery-type larvae injected with either vehicle or asc-C9 (n=3 with 5 beetles per group). **h.** Relative mRNA expression levels of UCP4 and PGC1α in recovery-type larvae injected with either vehicle or asc-C9 (n=3 with 5 beetles per group).

Earlier we showed that lower temperatures induce the production of a specific ascaroside, asc-C9, in *Monochamus* beetles in the fifth larval stage^16^. To investigate whether this ascaroside plays a role in thermogenesis, we compared the concentration of asc-C9 in beige-type and white-type larvae. This ascaroside was detected in beige-type larvae, but not in white-type larvae. In the field, the concentration of asc-C9 increased during the fall as temperatures decreased, reached a peak in January, and then fell from February to April, as the larvae transitioned from beige-type to recovery-type larvae (Fig. 2a). When beige- type larvae collected in the field were shifted from 4°C to 25°C in the lab, the concentration of asc-C9 in the larvae decreased and then disappeared (Fig. 2b). Furthermore, the asc-C9 concentration and UCP4 expression level were highest in the subcutaneous fat body of beige-type larvae (Fig. 2c).

Recovery-type larvae and white-type larvae treated with asc-C9 had higher survival rates and body temperatures, but low food intake (Fig. 2e, f, Fig. S2a, b, e). Asc-C9 injection increased the respiration rate and led to lower ATP production in recovery-type larvae, effectively making these larvae resemble beige- type larvae (Fig. S2a). The upregulation of lipid metabolism genes (Fig. S2d), lower TAG content (Fig. S2a), lower free fatty acids (Fig. S2c), smaller lipid droplets (Fig. 2d), and more mitochondrial DNA copy numbers (Fig. 2g) in recovery-type larvae after asc-C9 injection suggests asc-C9 induces lipolysis in beige-type larvae. UCP4 and PGC1α were up-regulated by asc-C9 in recovery-type larvae (Fig. 2h). In contrast, no differences were seen in the survival rate, body temperature, respiration rate, ATP content, or mitochondrial number between recovery-type larvae and recovery larval treated with a different ascaroside, asc-C5, which is one of the primary pheromones produced by pinewood nematode (Fig. S2e, f). Based on these results, it appears likely that thermogenesis in the subcutaneous fat body in beige-type larvae is induced by asc-C9.

#### **Ucp4** deletion blocks mitochondrial uncoupling by asc-C9

**Figure 3:**
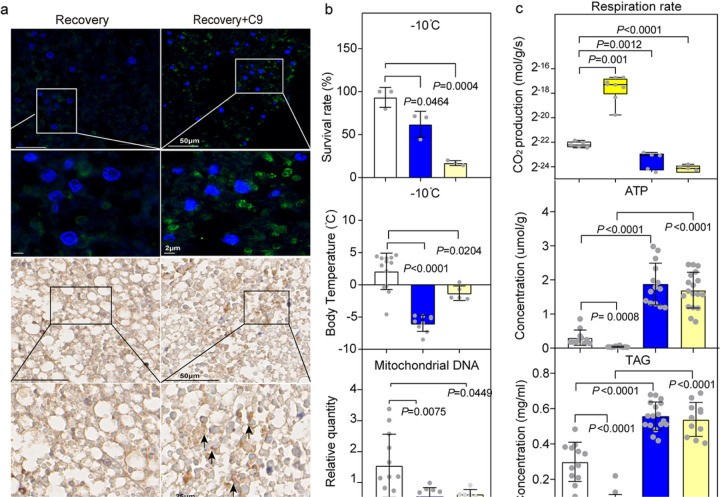
Mitochondrial uncoupling is mediated by asc-C9. **a.** Immunofluorescent detection and immunohistochemistry staining of UCP1 in sections of recovery-type larvae injected with either vehicle or asc-C9 (n=3 with 3 beetles per group). **b.** The survival rate, body temperature, and relative quantity of mitochondria DNA under −10°C in control, UCP4-depleted, and PGC1α-depleted beige-type larvae (n=5 with 5 beetles per group). **c.** Respiration rate, ATP content, and TAG level in dsGFP beige-type larvae, dsGFP beige-type larvae injected with asc-C9, UCP4-depleted beige-type larvae, and UCP4-depleted beige-type larvae injected with asc-C9 (n=3 with 10 beetles per group).

After injection with asc-C9, UCP4 is induced in recovery-type larvae, based on immunofluorescence (Fig. 3a). Disruption of UCP4 and PGC1α in beige-type larvae exposed to cold shock resulted in a significantly lower survival rate, slower cold shock recovery, reduced body temperature and increased mitochondrial number (Fig. 3b, Fig. S3a). PGC1α was confirmed to regulate the expression of UCP4 (Fig. S3b). Importantly, the decreased respiration rate and increased ATP and TAG content after UCP4 depletion in beige-type larvae were not reversed by asc-C9 injection (Fig. 3c, Fig. S3c). These results suggested that uncoupled respiration is induced by asc-C9 in beige-type larvae. Therefore, UCP4 is necessary for the asc-C9-induced mitochondrial uncoupling and thermogenesis.

### Asc-C9-induced thermogenesis via AKHR modulated lipolysis

To elucidate the mechanism whereby asc-C9 stimulates thermogenesis, the regulation of G protein- coupled receptors (GPCRs) was scrutinized in the RNA-seq data of beige-type larvae, recovery-type larvae and asc-C9-injected recovery-type larvae. There were 13 GPCR genes significantly upregulated in beige- type larvae and asc-C9-injected recovery-type larvae, including AKHR (adipokinetic hormone receptor), a GPCR that controls lipolisis (Fig. S4a, b). In insects, the AKHR pathway is associated with β-adrenergic receptor signaling^20^, which controls UCP-1-stimulated thermogenesis in brown adipose tissue in mammals^5^. AKHR was thus selected for in-depth investigation of its capacity to induce UCP4-dependent thermogenesis.

**Figure 4:**
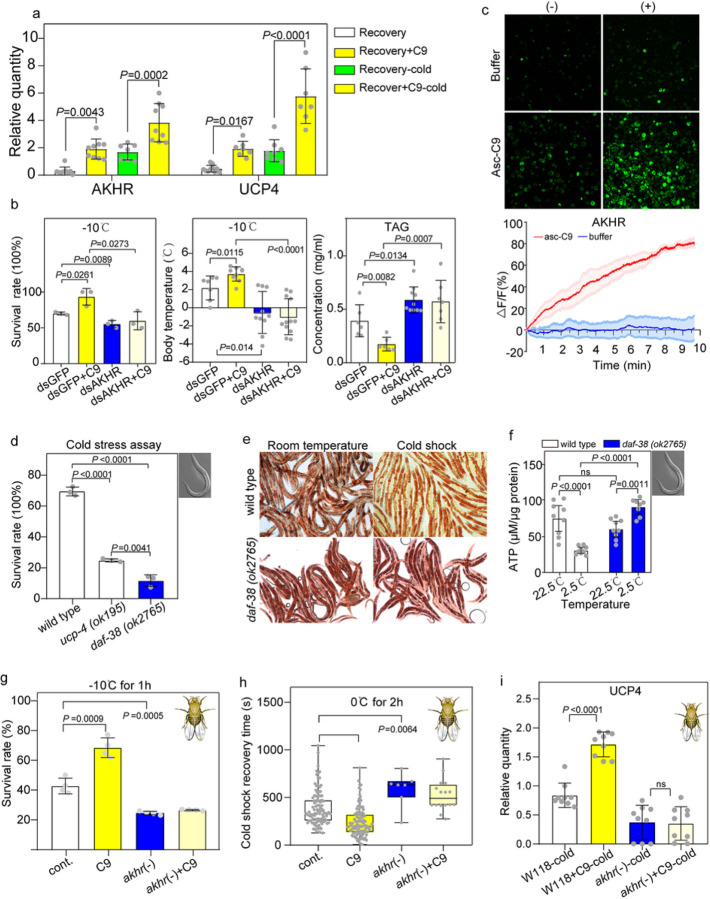
Asc-C9 stimulates energy expenditure through the AKHR receptor. **a.** The expression of AKHR and UCP4 after cold shock (at −10°C for 12h) in recovery-type larvae injected with either vehicle or asc-C9 (n=3 with 10 beetles per group). **b.** The survival rate, body temperature, and TAG concentration upon cold shock in dsGFP beige-type larvae, AKHR-depleted beige-type larvae, and AKHR-depleted beige-type larvae injected with either vehicle or asc-C9 (n=5 with 10 beetles per group). **c.** The Ca^2+^ imaging and fluorescence intensity curve of S2 cells with transfected *Monochamus* AKHR after asc-C9 added (n=3 with 3 per group). **d.** The survival of wild type, *ucp-4(ok195)*, and *daf-38(ok2765) C. elegans* in cold stress assay (n=3 with 3 per group). **e.** The fat levels of wild type and *daf-38(ok2765) C. elegans* at room temperature (22.5°C) and low temperature (2.5°C) (n=3 with 3 per group). **f.** The ATP level of wild type, *ucp-4(ok195)*, and *daf-38(ok2765) C. elegans* at room temperature (22.5°C) and low temperature (2.5°C) (n=9 with 3 per group). **g.** Survival rate after cold shock at −10°C for 1h of wild-type and *akhr-*mutant *D. melanogaster* fed with either vehicle or asc-C9 (n=3 with 20 flies per group). **h.** The recovery time after cold shock at 0°C for 2h of wild-type and *akhr-* mutant *D. melanogaster* fed with either vehicle or asc-C9 (n=3 with 20 flies per group). **i.** The expression of UCP4a after cold shock (at 0°C for 2h) of wild-type and *akhr-*mutant *D. melanogaster* fed with either vehicle or asc-C9 (n=3 with 3 flies per group).

Asc-C9 increased AKHR transcriptional abundance both under room temperature and cold stress (Fig. 4a). After AKHR depletion, the survival rate upon cold shock, cold shock recovery, and body temperature all decreased, while the TAG content increased. These effects were not reversed by asc-C9 injection, suggesting that the ascaroside mediates its effects via AKHR (Fig. 4b, Fig. S4c). Moreover, after AKHR depletion, the expression of UCP4 and PGC1α decreased and did not increase after asc-C9 injection (Fig. S4c). These results indicate UCP4 is regulated by the ascaroside and that this regulation occurs through AKHR.

Calcium imaging assays were conducted to examine the response of the AKHR to asc-C9. We monitored the change in Ca^2+^ concentration represented by fluorescence intensity (△F/F) of S2 cell transfected with GFP-tagged *Monochamus* AKHR after treatment with 300nM of asc-C9. The asc-C9 stimuli increased △F/F with a maximum change in fluorescent intensity of more than 60%. This result demonstrated that AKHR responds to asc-C9 (Fig. 4c).

Of the ascaroside receptors in *C. elegans*, insect AKHR shows strongest homology to *C. elegans* DAF-38 (Fig. S4d). DAF-38 is thought to bind to a relatively broad range of ascarosides^21^. We found that in *C. elegans*, the *daf-38* mutant is strongly sensitive to cold stress to a similar degree as the *upc-4* mutant, which was previously shown to be important for survival during cold stress (Fig. 4d)^22^. Moreover, the *daf-38* mutant stores more fat than wild-type worms in both room-temperature conditions and cold shock (Fig. 4e). Unlike wild type, the *daf-38* mutant did not show a decrease in ATP levels upon cold shock, suggesting that it was not responding to cold shock through mitochondrial uncoupling (Fig. 4f). These results indicate that DAF-38 may mediate a response to cold shock, involving lipolysis and UCP-4- mediated uncoupling, in *C. elegans*.

We further verified that the effects of asc-C9 are mediated via AKHR using the fly *Drosophila melanogaster akhr-* mutant. The survival rate of the *akhr-* mutant was lower than wild type under cold exposure (−10°C). The recovery time from cold shock at 0°C also increased significantly in the *akhr-* mutant relative to wild type (Fig. 4g). Asc-C9 enhanced the cold shock survival and decreased the cold shock recovery time in wild type, but not in the *akhr-* mutant (Fig. 4h). Asc-C9 upregulated the expression of UCP4a and AKHR in the fly under cold shock (0°C) in wild type, but not in the *akhr-* mutant (Fig. 4i).

### BAT activation and subcutaneous WAT browning by asc-C9 in mice

**Figure 5:**
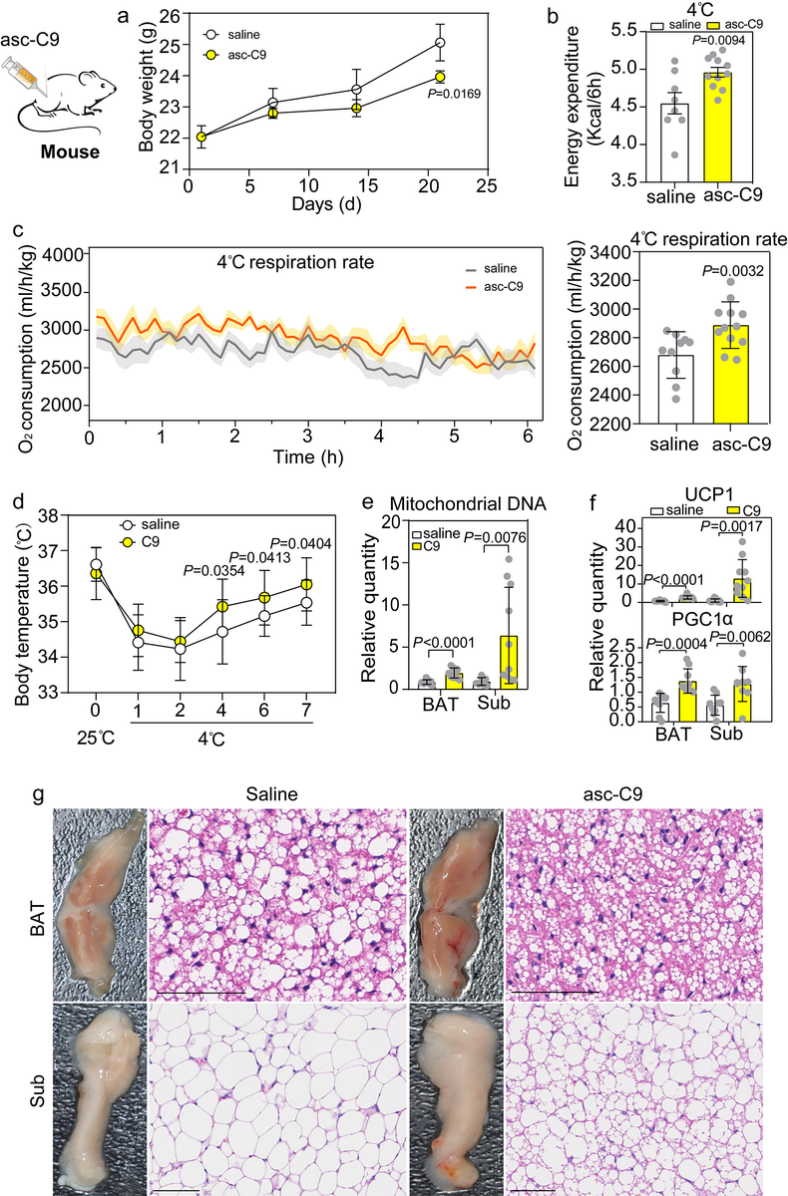
Ascaroside-C9 activates UCP1 in mouse BAT. **a.** The body weight at 25°C of mice injected with saline or 200μg /mouse asc-C9 for 1 month (n=3 with 10 mice per group). **b.** The energy expenditure at 4°C over 6h for mice injected with saline or 200μg /mouse asc-C9 every 2 days for 1 month (n=3 with 10 mice per group). **c.** The respiration rate at 4°C over 6h for mice injected with saline or 200μg /mouse asc-C9 every 2 days for 1 month (n=3 with 10 mice per group). **d.** The body temperature after shifting from 25°C to 4°C over 7h for mice injected with saline or 200μg /mouse asc-C9 for 1 month (n=3 with 10 mice per group). **e.** The morphology and hematoxylin-eosin staining of BAT and subcutaneous adipose tissue in mice injected with saline or 200μg /mouse asc-C9 for 1 month (n=3 with 10 mice per group). **f.** The relative mitochondrial DNA quantity in BAT and subcutaneous adipose tissue for mice treated with saline or 200μg /mosue asc-C9 (n=3 with 10 mice per group). **g.** The relative quantity of UCP1 and PGC1α in BAT and subcutaneous adipose tissue for mice treated with saline or 200μg /mosue asc-C9 (n=3 with 10 mice per group).

**Figure 6:**
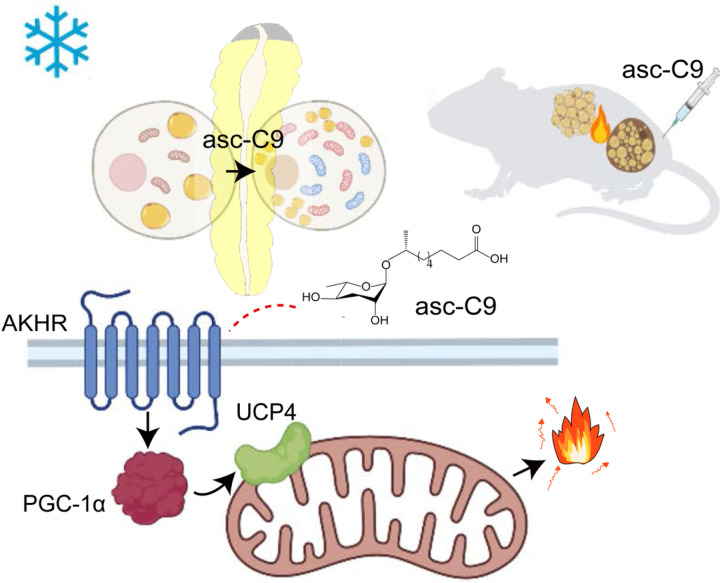
Schematic diagram of asc-C9 stimulated fat browning in *Monochamus* beetle and mouse.

In mice treated with asc-C9, the body weight decreased (Fig. 5a), the energy expenditure (Fig. 5b), respiration rate and body temperature under cold exposure at 4°C were elevated (Fig. 5c, d). The BAT and subcutaneous adipose tissue of mice displayed a distinctly darker brown color after injection with asc-C9 compared to controls (Fig. 5g). The BAT and subcutaneous adipose tissue showed large, clustered populations of cells with smaller multilocular lipid droplets, and also increased mitochondrial density after injection by asc-C9 (Fig. 5g, e). These results indicated that asc-C9 activated BAT and also induced the browning of WAT in the subcutaneous adipose tissue. Accordingly, thermogenic genes, such as Ucp1, were elevated in both brown and subcutaneous fat depots (Fig. 5f). These results show that asc-C9 can broadly induce mitochondrial biogenesis and fat thermogenesis in mice (Fig. 6). Interestingly, asc-C9 also reduced the lung tumor volume and tumor weight (Fig. S5).

## Discussion

Here, we demonstrate that an ascaroside (asc-C9), which was first found in nematodes and later in insects, can effectively induce thermogenesis in adipocytes. We have shown that the activity of this chemical signal as a novel fat-burning activator spans from insects (beetles and flies) to mice. Thus, this chemical signal is a promising tool to investigate the role of thermogenesis in cold tolerance, obesity/diabetes, and even tumour suppression.

Our work uncovers a new function for an ascaroside by showing that it likely acts as a hormone in the beetle and induces metabolic changes not only in the beetle, but also in other insects and mice. In nematodes, specific ascarosides promote the formation of the dauer larval stage in response to a stressful environment (high population density and low food) while other ascarosides influence a variety of behaviors. Ascarosides are thought to function primarily as pheromones and are secreted into the environment as chemical communication signals. Increasing evidence, however, suggests that ascarosides may also serve as signaling molecules inside the worm^23^. Previously, we showed that asc- C9 is produced by *Monochamus* beetle larvae where it suppresses larval development^16^. Here, asc-C9 was further found to trigger thermogenesis in the fat body as a cold-induced signaling molecule inside *Monochamus* beetle larvae.

The molecular mechanism of thermoregulation is conserved to a certain degree between nematodes, insects and small mammals. The fat body of beetles shares a similar thermogenic mechanism with the BAT of mammals to raise body temperature as a defense against cold stress. BAT activation is predominantly ascribed to the Gs-coupled family of GPCRs (ß-adrenergic receptor, GPR3, secretin receptor, glucagon receptor, GIP receptor, A2A receptor, melanocortin 2 receptor)^24–30^. AKHR is an important GPCR in insects that inhibits feeding frequency and promotes lipid metabolism^31,32^. Similar to the ß-adrenergic receptor, Gs-coupled AKHR assures rapid hormone-sensitive lipolysis^2,20^. Our work shows that AKHR in the beetle also plays an important role in inducing mitochondrial proliferation and uncoupling via UCP4 in order to improve cold tolerance. Interestingly, the *C. elegans* homolog of AKHR, DAF-38, which responds to a range of ascarosides, also appears to be important for cold tolerance, possibly through its effects on lipid metabolism.

Previous research has reported that insect UCP4 displays the highest homology to the unique mammalian UCP1, and UCP4 represents the ancestral UCP from which other UCPs evolved^8^. Our work shows that the expression of UCP4 and its transcription factor PGC1α in the fat body of the beetle has thermogenic activity. Thus, the function of UCP4 in insects may be similar to that of UCP1 in mammals.

In summary, we demonstrate that asc-C9 activates UCP-mediated thermogenesis through AKHR in insects and mice. Ascarosides may provide a novel therapeutic approach to activate thermogenesis, which is vital for the survival of animals under cold stress and also inhibits the development of tumours. The antitumour growth properties of asc-C9 in mice deserve further study.

## Experimental model and subject details

### Beetle collection and rearing

The beige-type larvae of *Monochamus alternatus* were collected from host trees of *Pinus massoniana*, in Nanjing, Jiangsu province from 2018 to 2022, when the fifth-stage larvae experience the overwintering period from mid-October to April. The beige-type larvae were reared in the laboratory with an artificial diet (200 g of sawdust, 15 g of agar, and 550 mL of distilled water) in a 10 mL tube in a climate chamber (10°C, dark, as 10°C maintains asc-C9 production and is the temperature the beetles experienced in the field). Fresh diet was provided every week. The white- type larvae of *M. alternatus* were reared on the same artificial diet in a climate chamber (25°C, dark). The recovery-type larvae of *M. alternatus* were reared at gradient heating in a climate chamber (4°C, 10°C, 15°C, 20°C, and 25°C for 7d respectively, in order to decrease asc-C9 production) and supplied with the same artificial diet. Cumulative food intake were detected each day.

### Fly models

*Drosophila* population w1118 (Bloomington stock number 5905) was used as a wild-type control. These fly lines were a gift from Professor Zhou Chuan’s laboratory at Institute of Zoology, Chinese Academy of Sciences. Flies were raised on standard medium at 25°C. *Drosophila* were reared from embryos in low-density bottles with standard medium containing 3.16% brown sugar, 6.32% glucose, 3.22% yeast, 1.06% agar, 7.77% cornmeal, 15% juice, supplemented with 1.75% methylparaben mix (10% methylparaben in ethanol), and 0.4% propionic acid. All percentages given in w/v except methylparaben mix and propionic acid given in v/v.

### Mouse models

The C57BL/6N Crl male mice of 6–8 weeks of age were purchased from Beijing Vital River Laboratory Animal Technology Co., Ltd (Beijing, China). The mice were kept at room temperature (24–26°C) in a humidity-controlled (45–65%) room with 12 h light/dark cycle. Standard normal chow diet and sterile water were given. Mice were randomly divided into 4–5 groups (n = 5–6/group) with average same weight (20–30g each). Grouped mice received intraperitoneally (i.p.) administered injection of asc-C9 (10mg/kg ≈ 200μg for each mouse) and normal saline every 2d for 4 weeks. All animal protocols in this study were approved by the Animal Care and Use Committee of the Institute of Zoology, CAS. Body weight were detected each day.

#### **Caenorhabditis elegans** models

*C. elegans* variety Bristol, strain N2 (wild type), *daf-38 (ok2765)*, and *ucp-4 (ok195)* mutant worms were grown at room temperature (20–23°C) on NGM agar plates and seeded with OP50 bacteria.

### Cell culture

The *Drosophila* Schneider 2 (S2) cells (RRID: CVCL_Z232) were a kind gift of the research group of Professor Zou Zhen, Institute of Zoology, CAS. *Drosophila* S2 cells were cultured in insect medium (Gibco) supplemented with 10% fetal bovine serum (FBS, Hyclone) and antibiotics (penicillin and streptomycin, Invitrogen) at 27°C.

### Methods

#### Ascaroside concentration determination, treatment and fat body collection

Quantitative determination of asc-C9 and asc-C5 content in the larvae of various types and treatments was carried out according to a previously developed method^16^. The concentrations were determined in larvae around the same weight (0.3–0.4g). The asc-C9 concentration in different tissues (epidermis, subcutaneous fatbody, perivascular fatbody, and midgut) was determined by dissecting the different tissues and extracting them in 1 mL of absolute ethanol at 25°C and shaking at 150 r.p.m for 24h. The extracts were purified through filter paper, lyophilized in a vacuum centrifugal concentrator (RVC2–18CD, Sigma), and resuspended in 400 μL of 50% (vol/vol) methanol in water. HPLC-MS was performed using an Agilent 1290 Series HPLC system equipped with an Agilent ZORBAX SB-C18 column (2.1×150 mm, 5μm particle diameter) connected to Agilent 6400 Series Triple Quadrupole spectrometer. Metabolite extracts were analyzed by HPLC-ESI-MS in negative and ion modes. The ascarosides were identified using a MS/MS screen for the precursor ion of *m/z* = 73.0. There were eight replicates for each experiment.

To test the effect of asc-C9 on the beetle’s cold survival, 5μL of different concentrations (30nM, 100nM, 300nM, 900nM) of asc-C9 were injected into the beetle around the same weight (0.3–0.4g), based upon the concentration of asc-C9 detected in beige-type larvae. 5μL of 900nM (0.045nmol, the same amount as the fifth-stage larval beetles in December) asc-C9 was injected into the abdomen of recovery-type larvae to measure the expression of UCP4. 24h after the injection, lipid droplets, respiration rate, ATP, TAG content, and relative mitochondrial DNA quantity were detected (lipid droplets were observed for 1–4d after asc-C9 injection, 24h showed the most difference). asc-C9 or asc-C5 were added to the artificial diets (0.45nmol asc-C9 or asc-C5 in every 10g artificial diets) of white-type and recovery-type larvae to test their effects on cold survival. The asc-C9 and asc-C5 were synthesized and tested as previously described^16^.

The larvae of *M. alternatus* were dissected under a stereo microscope (OLYMPUS SZX16, Japan), and the fat body was collected into a 1.5 mL RNase-free microcentrifuge tube, and the samples were quickly frozen in liquid nitrogen for use.

### Measurement of body temperature, mortality rate and recovery assay

#### Beetles

The thermogenic ability of *M. alternatus* was determined in the beige-type, white-type, and recovery-type larvae, when they experienced continuously increased ambient temperature from − 10°C for 10min, and then were transferred to 15°C, 20°C, and 25°C for 10min each. In addition, the body temperature was recorded in beige- and white-type larvae when they were put at 25°C for 30 min, and 25°C for 4d. The thermogenic ability was based on the difference between the ambient temperature and the body temperature.

The measurements of body temperature were recorded with a FLIR T620 camera, with a 480 × 640 pixel bolometric array (technical data available online at http://www.Flirthermography.co.uk/cameras/camera/1079/). The thermal images acquired by the camera were post-calibrated on the basis of the acquisition distance, mean infrared reflectance of the observed object, reflected temperature and air temperature by means of FLIR QuickReport freeware package (download page: http://www.flir.com/thermography/eurasia/en/content/?id= 11368).

In mortality experiments, beetle larvae from each treatment were exposed to − 10°C for 17h 30 min in incubator. After that, all larvae were placed in a climate chamber (25°C, light:dark = 12:12 h) to recover for 1d. Death was assessed by the absence of mandibular or body movement when larvae were stimulated with a needle. The cold shock recovery was determined by the frequency of the larval head swinging within 1 min. Nine to twelve individuals were tested in each treatment. Each treatment had three replicates.

#### Flies

To determine the cold survival of flies treated with asc-C9, w1118 flies (6–7 days old) were transferred to the medium containing 1uM asc-C9 and fed as the treatment group. Control group were raised without asc-C9. Two days later, flies were placed in the incubator at −10°C for 65min then returned to the incubator at 25 ° C for 1h. If the fly did not flutter or move, it was marked as dead. Ten individuals were tested in each treatment. Each treatment had three replicates.

#### Mouse

Mice were exposed to 4°C to test their cold endurance. Rectal temperatures were measured at 0, 30, 60, 90, 150 and 240 min using a digital thermometer (TH212, China) to represent the core body temperature. Mice had free access to water.

### CO_2_ respiration assay

#### Beetles

*M. alternatus* beetle larvae were placed individually into customized respiration chambers (10mL). The test container was installed in an incubator at 25°C ± 0.5°C, which is the most suitable temperature and cultivation environment for the insects. Each insect was allowed to acclimate for 10 min before testing. After testing, the insect were returned to the same conditions as before. The respiration rate was estimated by measuring carbon dioxide consumption using closed-system respirometry. The resulting carbon dioxide concentration (% CO_2_) of the samples was measured using an CO_2_ analyser (FOXBOX, Sable Systems, USA) and recorded every second using a data acquisition system (ExpeData, Sable Systems). Samples were set at a constant flow rate of 100 mL/min, using a Mass Flow Controller (MFC2), through a column containing drierite and then to the CO_2_ analyser. Before each trial, outdoor air was used to calibrate the analyser. The rate of CO_2_ release (RCO_2_, in mol/g/s) was calculated as: (the slope of CO_2_ (%/sec) × exact respirometry system volume (97.18 mL) × atmospheric pressure measured (kPa) × 1000)/ (100 × 298 (K) × 8.314 × 1,000,000 × insects’ weight (g)). The mean CO_2_ (VCO_2_, in mL/ s) was calculated as: (1,000,000 × 8.314 ×RCO_2_ × 298 (K) × insects’ weight (g))/ (1000 ×atmospheric pressure measured (kPa)). For mitochondrial drug feeding experiments, beetles were injected the following compounds for 24h: 5mM oligomycin (blocks ATP synthase), 2.5% stearate (induces mitochondrial uncoupling), 2mM GTP (inhibits mitochondrial uncoupling).

#### Mouse

Changes in carbon dioxide respiration of mouse were estimated by measuring the rate of oxygen consumption using an open-flow respirometry system (TSE, Germany). Dry the airflow pipe of the instrument thoroughly before use. The incurrent flow rate was 900 mL/min and the flow rate through each oxygen analyzer was 380 mL/min. The mice were placed in respiratory chambers individually (TSE, type I for mice, volume 2.6L). The chamber temperature was maintained at 4 ± 0.5°C for 8h. The mice can access to food and water during the measurements. The data were recorded and averaged every 10 s by a computer connected via an analogue-to-digital converter that converted the changes of air composition to digital signal (TSE system, Germany).

### Hematoxylin-eosin staining

Adipose tissues were fixed in 4% paraformaldehyde, processed sequentially in ethanol, xylene and embedded in paraffi n blocks, and then 5 μm thick slices were cut onto glass slides. After staining the tissue with hematoxylin and eosin (H&E), the coverslips were mounted with neutral resin. Tissue sections were observed by confocal microscopy (LSM 710; Carl Zeiss).

### Electron microscopy sections

Fresh larvae fat bodies were immersed and fixed in 2.5% glutaraldehyde, washed with 1xPBS solution, fixed with 1% osmic acid, dehydrated with 30%, and the 50% ethanol, and immersed in uranyl acetate solution at 4°C in the dark 12h. Tissues were again dehydrated with a gradient of ethanol (80%, 90%, 100%) from low to high level. After soaking the tissue in acetone for 20 min, it was soaked in different proportions of mixed embedding medium for 1 h, 3 h, and 5 h successively, then placed in the mold and sliced with an ultramicrotome, and finally stained with lead citrate and observed under a transmission electron microscope.

### Immunofluorescence

For immunofluorescence, larval *M. alternatus* were prepared as 7 μm frozen sections using a Leica CM1900 (Germany). The samples were kept at 37°C for 10 min, and were fixed with cold acetone for 10 min, then were washed 3 times for 5 min each with PBS. Then the samples were blocked with 0.1% Triton X-100 in 5% bovine serum albumin (BSA) for 1 h and further incubated with the same buffer containing UCP1 antibody (polypeptide antibody, dilution 1:100) overnight at 4°C. As reference gene, α-tubulin was used in this study. The samples were then washed five times for 5 min each with PBS and secondary antibody (dilution 1:200; Alexa Fluor 568 goat anti-rabbit IgG, Invitrogen) was applied for 30 min. The cellular nucleus was stained using 1 μg/mL DAPI (excitation at 358 nm, emission at 461 nm) for 10 min. Finally, the samples were mounted using anti-fade mounting medium (invitrogen) and analysed by confocal microscopy (Zeiss LSM710).

### Mitochondrial DNA copy number and Adenosine Triphosphate (ATP) Levels

Mitochondrial DNA copy number was assessed by qPCR with DNA as template using primers against COXII and normalized to RpL32. Mitochondria isolated from the fat body of *M. alternatus* larvae were treated according to the ATP content detection kit (BC0300) of Beijing Solarbio Science & Technology Co., Ltd. 0.625 μmol/mL standard solution was prepared for immediate use. An ultraviolet spectrophotometer was used to detect the absorbance at a wavelength of 340nm, and the absorbance values A1sample and A1standard of the sample and standard were recorded a 10s and the sample/standard was then quickly placed in an incubator at 25°C for 3min. The absorbance values A2sample and A2standard of the sample and standard were then determined at 3min, 10s, and the ATP level of mitochondria were determined by calculation:

$$\text{ATP level}(\mu \text{mol}/\text{g})=0.625\times {\text{(A2}}_{\text{sample}}{\text{-A1}}_{\text{sample}}\text{)}÷{\text{(A2}}_{\text{standard}}{\text{-A1}}_{\text{standard}}\text{)}÷\text{W=0}.625\times \Delta {\text{A}}_{\text{sample}}÷\Delta {\text{A}}_{\text{standard}}÷\text{W}$$

### Transfection of S2 cells and Ca^2+^ imaging

S2 cells were grown to approximately 80–90% confluence as observed under a light microscope. Cells were dislodged from the flask by washing with the media contained in the flask. In total, 1× 10^6^ S2 cells were suspended in 3 mL serum-free medium (SFX, Hyclone, SH30278.02, USA) in each well of a Nunclone six-well tissue culture plate (Corning Inc., Corning, NY, USA). Confluent cells (80–90%) were transiently transfected with 2.0 mg pMT-V5-HisA-AKHR using 8 mL of Cellfectin II reagent (Invitrogen, Carlsbad, CA, USA) in six-well plates according to the manufacturer’s instructions. The medium containing plasmid DNA and Cellfectin II was removed after incubation for 24 h. The cells were washed twice with fresh S2 medium and overlaid with 3 mL of fresh SFM.

After the cells were transfected for 48 h, the medium was removed and the cells were washed three times with Hank’s balanced salt solution (without Ca^2+^). The cells were subsequently cultured at 37°C in the dark for 30 min in the presence of 2 mM Fluo-4-AM (a final concentration of 10^− 6^ M) (Invitrogen, USA) and were stimulated by 300nM asc-C9. Calcium imaging was performed with a two-photon microscope. The luminescence assay was performed by measuring the intracellular Ca^2+^ mobilization of the transfected S2 cells. The luminescence caused by the intracellular calcium mobilization was measured for 20 s in half-second intervals by a TriStar2 LB 942 Multimode Reader (Berthold Technologies, Bad Wildbad, Germany). A concentration-response curve was calculated for asc-C9 by log fitting in Origin 8.6 (OriginLab, Northampton, MA). All experiments were conducted with three biological replicates.

### RNAi experiments

dsRNA was prepared using the T7 RNAi Transcription Kit (Vazyme) following the manufacturer’s instructions. Using a microsyringe, 30 nM of the corresponding dsRNA was injected into the middle segment of the back of beetle larvae, and samples were collected 3 d after injection. All primers used for making dsRNA are listed in Table S1.

#### Cold stress survival assay, Oil Red O (ORO) staining, and ATP analysis in C. elegans

To examine the survival of *C. elegans* under cold stress, 50–100 synchronized young adult stage worms were prepared. The synchronized egg lay method was performed by placing several young adult worms on a 6 cm agar plate seeded with OP50 bacteria and then removing the worms after 2 h. Once the progeny reached the young adult stage, they were transferred to a 2.5°C incubator for 24 h and then to a 25°C incubator for 1 h to recover. The worms were scored as alive if they responded to a gentle tap with a platinum wire pick. Oil Red O (ORO) staining was performed to assess fat storage. Synchronized worms were collected at the young adult stage and were washed with M9 buffer to remove bacteria. The samples were dehydrated and permeabilized with 40% 2-propanol for 3 min and were subsequently treated with ORO (3 mg/mL in 60% 2-propanol) and allowed to rotate in the dark at room temperature. After 2 h, the samples were washed with M9 to remove excess dye. For ATP determination, larvae were suspended in extraction medium (0.1 M NaOH, 0.5 mM EDTA) and incubated at 60°C for 20 min, then frozen at −80°C. Lysates were diluted and added to the assay solution (glycylglycine (250 mM, pH 7.4), EGTA (2 mM), MgCl_2_ (2 mM), BSA (0.4 g/L), DTT (7.5 mM)) with luciferin (0.015 mM) and luciferase (10 mg/mL). The reaction was initiated by addition of the samples and was allowed to incubate for 10 s. Light output was measured with a BMG Labtech CLARIOstar Plus Microplate Reader. ATP standards were run in parallel to determine relative ATP levels in each sample. A Bradford assay was performed to quantify the approximate amount of protein in each sample, relative to standards run in parallel.

### Statistical analysis

The comprehensive statistical analysis of the data utilized the software Excel 2010, SPSS 17.0. Data were expressed as means ± SD. Each treatment group uses the independent sample *t*-test comparison method for the significant difference analysis. Data graphing was done using the software GraphPad Prism 7.0.

## Data Availability

The data generated in this study are provided in the Supplementary information and Source Data file. Source data are provided with this paper.
